# Factors associated with non-attendance at scheduled infant follow-up visits in an observational cohort of HIV-exposed infants in South Africa, 2012–2014

**DOI:** 10.1186/s12879-019-4340-5

**Published:** 2019-09-16

**Authors:** Nobubelo Kwanele Ngandu, Debra Jackson, Carl Lombard, Duduzile Faith Nsibande, Thu-Ha Dinh, Vuyolwethu Magasana, Mary Mogashoa, Ameena Ebrahim Goga

**Affiliations:** 1 Health Systems Research Unit, South African Medical Research Council, Cape Town, South Africa; 2 UNICEF, New York, NY USA; 3 School of Public Health, University of the Western Cape, Cape Town, South Africa; 4 Biostatistics Unit, South African Medical Research Council, Cape Town, South Africa; 5 School of Public Health and Family Medicine, University of Cape Town, Cape Town, South Africa; 6 Division of Global HIV and Tuberculosis, Centers for Disease Control and Prevention, Center for Global Health, Atlanta, GA USA; 7 US Center for Disease Control and Prevention, Pretoria, South Africa; 8 Department of Paediatrics, University of Pretoria, Pretoria, South Africa

**Keywords:** HIV-exposed infants, Postnatal care, Missed visits

## Abstract

**Background:**

Since 2001 the South African guidelines to improve child health and prevent vertical HIV transmission recommended frequent infant follow-up with HIV testing at 18 months postpartum. We sought to understand non-attendance at scheduled follow-up study visits up to 18 months, and for the 18-month infant HIV test amongst a nationally representative sample of HIV exposed uninfected (HEU) infants from a high HIV-prevalence African setting.

**Methods:**

Secondary analysis of data drawn from a nationally representative observational cohort study (conducted during October 2012 to September 2014) of HEU infants and their primary caregivers was undertaken. Participants were eligible (*N* = 2650) if they were 4–8 weeks old and HEU at enrolment. All enrolled infants were followed up every 3 months up to 18 months. Each follow-up visit was scheduled to coincide with each child’s routine health visit, where possible. The denominator at each time point comprised HEU infants who were alive and HIV-free at the previous visit. We assessed baseline maternal and early HIV care characteristics associated with the frequency of ‘Missed visits’ (MV-frequency), using a negative binomial regression model adjusting for the follow-up time in the study, and associated with missed visits at 18 months (18-month MV) using a logistic regression model.

**Results:**

The proportion of eligible infants with MV was lowest at 3 months (32.7%) and 18 months (31.0%) and highest at 12 months (37.6%). HIV-positive mothers not on triple antiretroviral therapy (ART) by 6-weeks postpartum had a significantly increased occurrence rate of ‘MV-frequency’ (adjusted incidence rate ratio, 1.2 (95% confidence interval (CI), 1.1–1.4), *p* < 0.0001). Compared to those mothers with ART, these mothers also increased the risk of ‘18-month-MV’ (adjusted odds ratio, 1.3 (CI, 1.1–1.6), *p* = 0.006). Unknown infant nevirapine-intake status increased the rate of ‘MV-frequency’ (*p* = 0.02). Mothers > 24 years had a significantly reduced rate of ‘MV-frequency’ (*p* ≤ 0.01) and risk of ‘18-month-MV’ (*p* < 0.01) compared to younger women. Shorter travel time to health facility lowered the occurrence of ‘MV-frequency’ (*p* ≤ 0.004).

**Conclusion:**

Late initiation of maternal ART and infant prophylaxis under the Option- A policy and extended travel time to clinics (measured at 6 weeks postpartum), contributed to higher postnatal MV rates. Mothers older than 24 years had lower MV rates. Targeted interventions may be needed during the current PMTCT Option B+ (lifelong ART to pregnant and lactating women at HIV diagnosis) to circumvent these risk factors and reduce missed visits during HIV-care.

**Electronic supplementary material:**

The online version of this article (10.1186/s12879-019-4340-5) contains supplementary material, which is available to authorized users.

## Background

Optimal management of HIV in infants and children has been a challenge in low-middle income countries, resulting in missed opportunities for initiating timely treatment and averting adverse outcomes [1]. The reasons for sub-optimal care, despite well-structured guidelines for monitoring and follow-up, are complex and intertwined between the user and provider-related factors. Despite having made good progress in adult HIV treatment in South Africa, the country is still faced with a high loss to follow-up of HIV-exposed infants mainly during the high-risk breastfeeding period, with concomitant low coverage of infant HIV treatment [2, 3]. Data from a 2010 national survey in South Africa illustrates that 38% of early infant HIV testing opportunities are missed as a result of poor documentation of infant HIV exposure status or no maternal request for a test [4]. Other factors that contribute to unsatisfactory HIV care in children include poor parental uptake of HIV services, fear of stigmatization, limited knowledge about the modes of vertical HIV transmission (MTCT), young maternal age and low socioeconomic status [4–6].

Notwithstanding improved prevention of MTCT (PMTCT) policies from 2002 until 2015, the infant rapid HIV test at 18 months continues to mark the end point of the PMTCT programme, serving as a measure of PMTCT effectiveness [7]. Additionally, since 2005 the Integrated Management of Childhood Illness (IMCI) strategy, recommends that all infants, regardless of HIV exposure status, receive monthly routine growth monitoring and care (well-baby visits) during the first year of life, and every 3 months thereafter. There is little data on factors associated with non-attendance at scheduled child health care visits in the context of HIV.

We aimed to measure non-attendance at intermittently scheduled study follow-up visits, and at the final 18-month exit visit, amongst HIV-exposed uninfected (HEU) infants enrolled in a nationally-representative survey to measure postnatal PMTCT effectiveness. We also sought to identify factors associated with non-attendance at these visits to shed light on possible interventions to improve postnatal follow-up.

## Methods

### Study data and aims

Secondary analysis of data drawn from a nationally representative observational cohort study of HEU infants and their primary caregivers was conducted. The main study, conducted between October 2012 and September 2014, aimed to measure postnatal mother-to-child transmission of HIV (MTCT) between 6 weeks and 18 months postpartum and these primary results are being presented in a separate manuscript. The cohort was recruited from 6 weeks postpartum cross-sectional survey conducted in 2012 whose methods have been previously published [8]; briefly, the study was conducted at public primary health care clinics and community health centres nationwide offering immunisation services. A nationally-representative sample of facilities was selected through a multistage probability proportional to size sampling approach. HIV Enzyme-linked immunosorbent assay (EIA) was used to confirm infant HIV exposure at 6 weeks of age. Infants who were HIV-exposed but not HIV PCR positive (i.e., HEU infants) at 6 weeks old were eligible for postnatal follow-up. Ethics approval for the study was granted by the South African Medical Research Council Ethics Committee, and approval was also obtained from the Centers for Disease Control and Prevention. All caregivers signed informed consent. The 6 week and 18 month HIV tests in this study were routine tests. A final sample of 2650 eligible caregiver-infant pairs who provided signed informed consent to take part in the cohort study was used in this secondary analysis. The cohort was followed up from October 2012 until September 2014. Participants were given study inconvenience allowances at each visit of USD$2.50 in October 2012, increased to USD$6.00 in January 2014. PMTCT Option A policy (maternal AZT plus infant ARV prophylaxis to prevent MTCT and infant prophylaxis until 1 week after cessation of breastfeeding) was in use in South Africa during the start of the study to March 2013. Subsequently, PMTCT Option B (maternal triple ARV prophylaxis to prevent MTCT and infant prophylaxis for the first 4–6 weeks regardless of feeding status) was adopted in April 2013 and was in use during the remainder of the study period [9].

The recruited HEU infants and their caregivers were followed up at 3, 6, 9, 12, 15 and 18 months post-partum. Data collectors attempted to coincide these study follow-up visits with routine follow-up visits during the same time interval and drew blood for infant HIV testing at each of the visits. Socio-demographic background information were collected at 6 weeks postpartum (baseline). Since the primary outcome for the cohort study was HIV incidence and death in HIV-exposed infants, the length of follow-up for each infant was determined by one of the following three scenarios: (i) until study end-point at 18 months postpartum if they remained HIV negative; (ii) up-to-the time point of infant seroconversion where after they presented for one exit interview to assess access to care or lastly, (iii) up-to-the time of death if they died before the study end-point or before HIV seroconversion.

This secondary analysis focusses on non-attendance of the six scheduled postnatal care study visits (at 3, 6, 9, 12, 15 and 18 months postpartum) and non-attendance of the 18-month postpartum visit.

### Describing patterns of ‘missed visits’

We studied non-attendance at scheduled visits, operationalised as ‘missed visit’, (abbreviated here as MV), for an infant who was eligible for that visit. Infants eligible for a visit were those enrolled at baseline who were alive and free of HIV at the previous visit. Repeated MV was observed for some participants; therefore, we also present simple proportions with 95% confidence intervals (CI) of MV at each follow-up study time point, i.e., per time-point MV.

Two scenarios of MV were of primary interest: The first, (i) ‘MV frequency’ defined as the number of MV across a participant’s exposure time over the study period. Exposure time is simply the number of months linked to scheduled eligible study visit points up to the end of the study (18 months postpartum) or time of death or HIV-infection. In this case, MV was a count variable, counting the number of missed eligible visits from 0 (attended all of them) through to 6 (was eligible for all study visits and never attended any of them). Exposure time ranged from 1 (if death or HIV-infection occurred at the 3-month point) to 6 (if either HIV-infection or death occurred after 18 months or never occurred during the study period. The second, (ii) 18-month MV, was a binary variable of failure to attend or not among those still eligible to attend this visit. The 18-month MV was of special interest because this visit was scheduled to coincide with the routine 18-month postpartum rapid HIV testing of the enrolled infant and the exit point from the PMTCT programme.

### Independent factors tested for association with MV

Purposively selected baseline socio-demographic characteristics, and health-care information was assumed to have some direct or indirect influence on the uptake of scheduled visits. The independent variables were evaluated for potential association with MV including relationship between the caregiver and the infant at the six-week enrolment, mother’s age, mother’s highest education level, marital status. We also evaluated whether infant was ever breastfed, disclosure of maternal HIV status to family or friends, ever facing discrimination due to HIV status, knowledge of MTCT modes, maternal ART use at 6 weeks postpartum, whether infant had been given nevirapine after birth, infant birth weight, infant hospitalization, means of transport used and time taken to access the facility, socio-economic status (SES) ranking and province. SES was extracted from a calculation using the full cross-sectional sample available at 6 weeks postpartum. In the full cross-sectional dataset, SES was calculated using principal component analyses from household characteristics (which included the type of housing, sanitation, water and fuel), household possessions (such as TV, stove, radio), any food shortage and source of income [10]. A Chi-squared test was used in the descriptive statistics of these independent variables at baseline and by ‘per time-point MV’.

None of the chosen independent variables had more than 10% missing data. Of those with some missing data, if excluding missing data changed the 95% CI of observed estimates on known categories of the variable, then an additional category for ‘unknown’ responses was added to the variable in the statistical and descriptive analyses in order to minimise deviation from actual observed estimates and to keep the survey structure and sampling weights accurate on the rest of the data.

### Identifying factors associated with MV

Two sets of analyses were conducted, to assess factors associated with ‘MV frequency’, and ‘18-month MV’, separately.

To assess the former, a regression model for a count outcome variable allowing varying exposure time periods was most suitable for the data. However, the assumption of a Poisson regression model for a count outcome (i.e., data are not overly dispersed), was not met. Considering that the outcome was expected to have zeros from participants who missed all their eligible visits (exposed), a zero-inflated binomial regression model was used to confirm whether the data were zero-inflated. The assumption of zero-inflation was rejected (z = 6.44, *p* < 0.0001). The final model used for identifying predictors of MV frequency was, therefore, a negative binomial regression, with survey set functions and exposure times specified [11, 12]. Bivariate analyses were first run between each independent factor and the primary outcome and a Wald test (corrected for survey structure and including the exposure time variable) performed to see if the coefficients were significantly non-zero using a *p*-value cut-off of < 0.25. Independent factors with a Wald test *p* < 0.25 were then included in a multivariable analysis model. Those factors which had overall non-significant Wald tests but with *p* < 0.05 for the coefficients of sub-categories were then included in the multivariable model and retained if they caused a large change (a shift of the 95% CI) on the coefficients of any variables which were already in the model.

The final multivariable model included seven of the 15 independent variables; maternal age, caregiver status, the disclosure of maternal HIV status, infant ever being on nevirapine, the mother being on ART, common means of transport and province. Adjusted point estimates are presented here.

To identify factors associated with ‘18-month MV’, a logistic regression model, with survey settings specified, was used. For ‘18-month MV’, ten factors (maternal age, education level, whether infant ever breastfed, disclosure of maternal HIV status, being discriminated against, knowledge of MTCT modes, mother being on ART at 6 weeks, infant having taken nevirapine by 6 weeks, SES and province) were eligible for inclusion in the final multivariable model.

All analyses were performed in STATA SEv13. Sampling weights were applied to all reported proportions and statistical analyses to adjust for sample ascertainment in the original cohort and non-consent for postnatal follow-up amongst all those eligible for postnatal enrolment.

## Results

### Baseline characteristics of the cohort

The majority of caregivers at 6 weeks postpartum (baseline) were the infants’ mothers (97.6%), with a mean maternal age of 29 years (interquartile range 24;33 years). Three age-groups were used for analyses in the enrolled sample, adolescents and young adults (< 14–25 years old, 23.3%), 25–34 years of age women (58.2%) and older women (at least 35 years old), Table 1. Most caregivers had achieved at least 7 years of schooling (81.0%) and were either single, widowed or divorced (78.0%). Only 27.3% of infants had never been breastfed during the first 6 weeks of life. Seventy-seven percent of mothers had disclosed their HIV status to family or friends, but only 8.6% reported ever feeling discriminated against due to their HIV status. Eighty-nine percent of caregivers knew at least one mode of MTCT; 52.3% of mothers were on ART and 85.6% of infants had received nevirapine prophylaxis by the first 6 weeks of life. Overall 13.9% of enrolled infants had low birth weight and 5.1% had been hospitalised by 6 weeks postpartum. Although 4 % of women used their cars to travel to the clinic, 77.1% reached the health facility in under 30 min by either walking or using public transport.

**Table 1:** Characteristics of the study population at baseline (*N* = 2650)

Characteristic	n	weighted % (95% CI)
Caregiver		
Mother	2607	97.6 (96.7–98.3)
Other	43	2.4 (1.7–3.3)
Mother’s age~		
13–24 years	681	23.3 (21.5–25.2)
25–34 years	1480	58.2 (56.0–60.3)
35–50 years	482	18.1 (16.5–19.7)
Education~		
Grade 7 and below	525	18.8 (17.3–20.5)
Grade 8 and above	2121	81.0 (79.4–82.5)
Marital status~		
Married/cohabit	648	22.0 (20.4–23.7)
Single/widow/divorced	2000	78.0 (76.3–79.6)
Ever Breastfed~		
Yes	1880	72.7 (70.9–74.5)
No	702	27.3 (25.5–29.1)
Disclosure of HIV mother’s status^a^		
Yes	2087	76.5 (74.6–78.3)
No	455	17.4 (15.9–19.1)
Unknown	108	6.1 (5.0–7.4)
Discriminated against regarding HIV status^a^		
Yes	220	8.6 (7.5–9.9)
No	2322	85.3 (83.6–86.9)
Unknown	108	6.1 (5.0–7.4)
Knows MTCT modes^a^		
Yes	2327	88.6 (87.2–89.8)
No	87	3.3 (2.6–4.2)
Unknown	236	8.2 (7.1–9.3)
Mother on ART by 6 weeks^a^		
Yes	1445	52.3 (50.2–54.4)
No	1083	41.1 (39.1–43.2)
Unknown	122	6.6 (5.5–7.9)
Infant on NVP by 6 weeks^a^		
Yes	2329	85.6 (84.0–87.1)
No	114	4.5 (3.7–5.5)
Unknown	207	9.9 (8.5–11.3)
Infant birth weight~		
< 2.5 kg	374	13.9 (12.5–15.4)
2.5 kg +	2208	86.1 (84.6–87.5)
Infant hospitalised~		
Yes	123	5.1 (4.2–6.1)
No	2525	94.9 (93.9–95.8)
Travel to facility		
Own car	99	3.6 (2.9–4.4)
Walked < 30 min	1182	41.4 (39.4–43.4)
Walked > 30 min	283	10.4 (9.2–11.8)
Public transport < 30 min	872	35.7 (33.7–37.8)
Public transport > 30 min	214	8.9 (7.7–10.2)
SES^b^		
Highest	557	21.1 (19.4–22.8)
High	459	16.9 (15.4–18.5)
Middle	540	19.9 (18.4–21.6)
Low	543	20.5 (18.9–22.3)
Lowest	551	21.5 (19.9–23.3)
Province		
WC	246	6.5 (5.7–7.4)
NW	221	6.9 (6.0–7.8)
NC	84	1.5 (1.2–1.8)
MP	315	9.6 (8.6–10.7)
LP	266	9.3 (8.2–10.5)
KZN	442	26.8 (24.8–28.9)
GP	524	24.3 (22.5–26.2)
FS	275	5.2 (4.6–5.9)
EC	277	10.0 (8.9–11.2)

^a^a category for missing data was included. ~less than 3% missing data

^b^SES was calculated using all the data enrolled in the original cross-sectional survey at 6 weeks. South African provinces- WC-Western Cape, NW-North West, NC-Northen Cape, MP-Mpumalanga, LP-Limpopo, KZN-KwaZulu Natal, GP-Gauteng, FS-Free State, EC-Eastern Cape

### Patterns of MV per time-point

The lowest levels of MV per time point were at 3 and 18 months postpartum (32.7, 95% CI 30.8–34.7, and 31.0, 95% CI 29.0–33.0, respectively), (see Additional file 1 for baseline characteristics by patterns of MV per time point). For 6, 9, 12 and 15 month visits, MV ranged between 36.0 and 37.6% with no significant differences between the four-time points. Overall, the weighted percentages of MV appeared to differ significantly (*p* < 0.05) by type of caregiver who brought child at baseline, mother’s age, disclosure of mother’s HIV status to family or friends, discrimination, maternal ART, infant prophylaxis, means of travel to facility and province at which a participant resided.

A significantly higher proportion of 3 and 6 month visits were missed by infants brought by caregivers who were not their mothers at baseline (at 3 months = 70.2% versus 31.8% and at 6 months = 62.2% versus 35.8%, *p* < 0.0001 and *p* = 0.002 respectively). Differences in MV per time-point by maternal age were measured at 6, 12, 15 and 18 months postpartum. The youngest age-group of women, in particular, tended to have higher percentage of MV compared to the two older age-groups. MV at all time points except 18 months was significantly less in mothers who disclosed their HIV status at baseline. In the case of discrimination, significant differences were seen at 3, 6 and 9 months, where the lowest proportions of MV were among women who reported not being discriminated against due to their HIV status and the highest proportions among those with unknown responses. Lower uptake of infant nevirapine or maternal ART was related to MV at 3, 6 and 9 months and at all time points, respectively. Additionally, MV was highest among those with missing information about infant nevirapine or maternal ART. A significant difference in MV by mode of transport to the clinic was measured at the 15-month point (*p* = 0.02), and the highest MV was among women who used their cars and lowest amongst all those who walked or used public transport. There was no significant difference in MV by SES ranking, but MV differed significantly by province at all time points.

### Factors associated with ‘MV-frequency’

In total, 58.8% (95% CI 56.7–60.8) missed at least one scheduled follow-up visit (Table 2). The frequencies of missed visits by a number of scheduled visits, i.e., a breakdown of the MV-frequency, are shown in Additional file 2. Results from the negative binomial regression analyses adjusted for exposure time, mother’s age, type of caregiver interviewed at 6 weeks, maternal ART use at 6 weeks postpartum, whether infant had been given nevirapine after birth, disclosure of maternal HIV status, means of transport used and time taken to access the facility and province, demonstrated that the adjusted incidence rate ratio (aIRR) for‘MV-frequency’ tended to be significantly lower among older women (> 24 years of age) compared to adolescents and young adults (13–24 years old) and among those who had quick access to health facilities (less than 30 min) by either walking or using public transport compared to those who owned cars, but higher amongst mothers who were not on ART by 6 weeks postpartum and caregivers who did not know about infant intake of nevirapine (Table 2). To confirm whether young maternal age could be influencing delayed uptake of ART (i.e. not by 6 weeks post-partum), we included an interaction term of the two to the model but this effect was not significant and thus excluded from the final model. Differences between provinces were also evident.

**Table 2:** Baseline factors associated with ‘MV frequency’

Determinant	Exposed at Baseline (N)	MV frequency ≥ 1 n (weighted %)	Adjusted IRR	95% CI	*p*-value
ALL ->	2650	1548 (58.8)			
Caregiver					
Mother	2607	1514 (58.4)	ref		
Other	43	34 (75.2)	0.8	0.6–1.2	0.251
Mother’s age^a^					**0.01**
13–24 years	681	447 (66.1)	ref		
25–34 years	1480	848 (56.8)	0.9	0.8–1.0	**0.01**
35–50 years	482	248 (53.5)	0.8	0.7–1.0	**0.012**
Disclosure of mother’s HIV status					0.128
Yes	2087	1144 (54.5)	ref		
No	455	305 (66.7)	1.1	1.0–1.3	0.057
Unknown	108	99 (89.6)	0.8	0.4–1.7	0.596
Mother on ART by 6 weeks					**0.0003**
Yes	1445	769 (53.1)	ref		
No	1083	668 (61.1)	1.2	1.1–1.4	**< 0.0001**
Unknown	122	111 (89.2)	1.8	0.9–3.5	0.083
Infant on NVP by 6 weeks					**0.025**
Yes	2329	1331 (56.4)	ref		
No	114	68 (62.2)	1.2	1.0–1.4	0.118
Unknown	207	159 (77.7)	1.4	1.1–1.8	**0.02**
Travel					**0.012**
Own car	99	70 (72.3)	ref		
Walked < 30 min	1182	674 (58.6)	0.7	0.6–0.9	**0.002**
Walked > 30 min	283	173 (59.1)	0.8	0.6–1.0	0.068
Public transport < 30 min	872	498 (56.8)	0.7	0.6–0.9	**0.004**
Public transport > 30 min	214	133 (61.5)	0.9	0.7–1.1	0.231
Province					**< 0.0001**
WC	246	134 (56.7)	ref		
NW	221	112 (51.1)	1.2	0.9–1.5	0.305
NC	84	38 (46.3)	0.6	0.4–0.9	**0.006**
MP	315	193 (62.3)	1.3	1.0–1.7	**0.025**
LP	266	199 (73.8)	1.9	1.4–2.4	**< 0.0001**
KZN	442	226 (51.8)	1.3	1.0–1.6	0.103
GP	524	327 (63.7)	1.7	1.3–2.2	**< 0.0001**
FS	275	171 (62.4)	1.6	1.3–2.1	**< 0.0001**
EC	277	148 (55.1)	1.2	0.9–1.6	0.333

MV- missed visits at exposed postnatal care visits. IRR- incidence rate ratio. Significant *p*-values for the incidence rate ratio are in bold face

^a^variable has missing data of< 5%. All proportions were adjusted for survey sampling weights (W %). South African provinces- WC-Western Cape, NW-North West, NC-Northen Cape, MP-Mpumalanga, LP-Limpopo, KZN-KwaZulu Natal, GP-Gauteng, FS-Free State, EC-Eastern Cape

### Factors associated with ‘MV at 18 months’

After adjusting for all other independent factors in the final multivariable model, no maternal ART use by 6 weeks post-delivery significantly increased ‘MV at 18 months’ (adjusted OR (aOR) 1.3 (1.1–1.6), *p* = 0.006), whilst older maternal age significantly protected against MV at the 18-month visit point (aOR 0.7 (0.5–0.9), *p* = 0.001 for 25–34 year olds and *p* = 0.011 for 35 years old and older mothers) (Table 3). All provinces except for Northern Cape, had significantly higher odds of MV at 18 month visit point compared to the Western Cape Province. This model was controlled for maternal education; infant ever breastfed, the disclosure of maternal HIV status, having been discriminated against, knowledge of MTCT modes, the infant having taken nevirapine and SES.

**Table 3:** Baseline factors associated with ‘MV at 18 months’

Determinant	N	MV at 18 month time point: n (weighted %)	adjusted OR	95% CI	*p*-value
All	2555	767 (31.0)			
Mother’s age					
13–24 years	655	240 (37.9)	1.0	Ref	
25–34 years	1431	403 (28.6)	0.7	0.5–0.9	**0.001**
35–50 years	462	122 (28.0)	0.7	0.5–0.9	**0.011**
Education					
Grade 7-	501	167 (34.2)	1.0	Ref	
Grade 8+	2050	599 (30.3)	0.8	0.6–1.1	0.174
Ever Breastfed					
Yes	1811	558 (32.0)	1.0	Ref	
No	680	188 (28.2)	0.9	0.7–1.2	0.623
Disclosure of HIV mother’s status					
Yes	2009	578 (29.6)	1.0	Ref	
No	444	154 (35.6)	1.2	0.9–1.5	0.247
Unknown	102	35 (35.7)	0.7	0.1–3.2	0.601
Discriminated against					
No	2241	677 (85.5)	1.0	Ref	
Yes	212	55 (8.6)	0.7	0.4–1.1	0.093
Unknown	102	35 (6.0)	1.2	0.8–2.0	0.398
Knows MTCT modes					
Yes	2239	663 (30.5)	1.0	Ref	
No	86	24 (32.1)	1.1	0.6–1.9	0.762
Unknown	230	80 (36.3)	1.2	0.8–1.8	0.381
Mother on ART by 6 weeks					
Yes	1394	362 (26.9)	1.0	ref	
No	1045	364 (35.4)	1.3	1.1–1.6	**0.006**
Unknown	116	41 (36.3)	1.9	0.4–8.0	0.396
Infant on NVP by 6 weeks					
Yes	2251	664 (30.1)	1.0	ref	
No	105	36 (40.3)	1.5	0.9–2.5	0.091
Unknown	199	67 (34.7)	1.2	0.7–2.2	0.437
SES					
Highest	540	151 (28.6)	1.0	ref	
High	448	111 (28.8)	1.0	0.7–1.5	0.906
Middle	522	155 (28.9)	1.0	0.7–1.3	0.857
Low	523	174 (33.8)	1.1	0.8–1.6	0.415
Lowest	522	176 (34.4)	1.1	0.8–1.6	0.512
Province					
WC	239	32 (13.5)	1.0	Ref	
NW	213	68 (32.3)	2.8	1.6–4.8	**< 0.0001**
NC	82	6 (7.2)	0.4	0.1–1.4	0.175
MP	299	93 (32.0)	2.7	1.5–4.6	**0.001**
LP	252	97 (37.0)	3.5	2.0–6.0	**< 0.0001**
KZN	433	144 (33.3)	3.0	1.8–5.0	**< 0.0001**
GP	510	151 (31.5)	3.1	1.8–5.4	**< 0.0001**
FS	265	106 (39.6)	4.1	2.4–7.2	**< 0.0001**
EC	262	70 (26.4)	2.1	1.2–3.8	**0.014**

MV- non-attendance at scheduled postnatal care visits. Significant *p*-values for the odds ratio are in bold face. All proportions were adjusted for survey sampling weights (W %). South African provinces- WC-Western Cape, NW-North West, NC-Northen Cape, MP-Mpumalanga, LP-Limpopo, KZN-KwaZulu Natal, GP-Gauteng, FS-Free State, EC-Eastern Cape

## Discussion

Using data from a nationally representative observational cohort study which aimed to synchronise study follow-up visits for HEU infants with their recommended or planned routine postnatal follow-up visits where possible, we demonstrate that, ~ 59% of HEU infants did not attend one or more scheduled visits between 3 and 18 months postpartum. The lowest MV occurred at 3 and 18 months where just under a third of participants did not attend. Uptake of the 3 months visit could have been influenced by either the 14-weeks immunization uptake and/or that it was the first follow-up after enrolment [13]. Better uptake of the 18 months visit was probably linked to the fact that this is a major PMTCT end point visit and an immunization time point. Furthermore, study inconvenience allowances increased from USD$2.50 to USD$6.00 to cover participant travel and time costs, at eight calendar months before the study closure. The change in allowance amount could have motivated for better attendance thereafter at which most upcoming visits were for the 15 and 18-month points. It is also likely that maternal uptake of infant postnatal care is better when postnatal visits are fixed to specific activities such as vaccination rather than being labelled as a ‘general baby check-up’. It is also important to note that the rate of non-attendance could be lower under routine settings because participants were given inconvenience allowances during these study visits, which could have motivated some to return for follow-up.

The low uptake of study visits after 3 months is consistent with what has been observed in routine settings of other African countries. A study by Godfrey et al. (2015) for example, found extremely low retention rates for both mothers and infants between 4 and 12 months postpartum in Kenya, Malawi and Swaziland [14]. Sub-optimal retention in care has also been a challenge across different PMTCT regimen eras indicating that improving the regimen alone is not a solution but new and innovative strategies to promote better uptake of HIV care are also needed [15–18].

Not receiving maternal ART by 6 weeks postpartum was significantly associated with non-attendance regardless of how it was defined. Most importantly it was associated with missing the 18-month visit, which is a critical PMTCT visit as, in most countries, the 18-month HIV test is used to measure the final outcome of PMTCT interventions. Data collection for this study began when Option A regimen was in use during pregnancy and ended when the local policy had moved to Option B. For both Option A and Option B; ART was initiated in pregnant women with a CD4 count of 350 and less. The results seen here could reflect the differences in women’s attitudes then, between women whose lives were deemed to be at risk and hence on ART and the rest who otherwise probably did not find it necessary to attend as many visits. Unknown infant nevirapine-intake status at 6 weeks was associated with poor uptake of postnatal visits. This could reflect general neglect of child health issues and requires continued education and encouragement of caregivers during routine child health care visits. When looking at the breakdown of proportions of MV per time-point (see Additional file 1), the group with unknown infant nevirapine-intake had much higher MV until the 9 months time-point, and this difference was significant. From 12 months onwards, there were no longer significant differences in MV proportions between those who were on infant prophylaxis at 6 weeks, those who were not on infant prophylaxis at 6 weeks, and those who did not know the prophylaxis intake history. This finding, could mirror a change from the use of Option A regimen (cessation of infant prophylaxis at 6 weeks post cessation of breastfeeding) to Option B (infant prophylaxis for 6 weeks postpartum). That is, while Option A was active, infant prophylaxis continued during breastfeeding and therefore promoted infant visits to healthcare, to collect infant nevirapine, until breastfeeding cessation. Yet after Option B was implemented, infants were not required to be on nevirapine prophylaxis beyond 6 weeks postpartum, and thus not required to attend health facilities for HIV-care and prophylaxis. The influence of nevirapine intake on postnatal visits was therefore lost with Option B regimen. The significant results from the regression analyses therefore show that the strength of association between infant nevirapine prophylaxis intake and MV rates during the era of Option A was very strong.

Mothers older than 24 years, compared to adolescent and younger mothers were significantly associated with fewer MV in both definitions of MV. This finding is not surprising, given that provision of appropriate care for adolescents and young mothers have been identified as a critical gap in many countries [19–23]. Some earlier studies have consistently shown better awareness and uptake of HIV care among older women across different countries [23–25], and thus appropriate interventions for the younger age group are needed urgently. Interventions focussing on creating friendly attitudes and safe environments for adolescent-mothers in public healthcare facilities, aiming at making them feel as comfortable as older women in healthcare facilities, are needed urgently.

It was interesting to find that participants who took less time traveling to healthcare facilities, within 30 min, by walking or using public transport, had better outcomes compared to those who owned cars. The latter were not different from public transport users or walkers who took longer than 30 min to reach the facilities. Although it is not clear why those who owned cars performed poorer than the second group, the findings show that shorter time spent travelling to healthcare facilities by walking or public transport is an important factor for achieving good retention in care among mothers and their young children. This finding of better uptake among those with quicker access to facilities is not unique to South Africa. Accessibility to health facilities, in particular, availability of transport, transport costs and longer distances is a known obstacle to uptake and retention in care in other African settings [26, 27]. Investing in sustainable mobile clinics for the remote settings could facilitate uptake of health care among communities living far from healthcare facilities.

Significant differences between provinces were also observed for both types of MV, raising the need to investigate province-specific barriers.

We did not find any association between missing postnatal study visits and knowledge of MTCT modes or discrimination and disclosure of maternal HIV status in contrast to what has been reported by other earlier studies [28]. The findings related to disclosure and discrimination, in particular, are probably because disclosure was largely towards trusted close family or friends only, thus did not affect uptake of health-related care in any way. Also, a majority of the participants (three-quarters of the sample) had disclosed their HIV status and only less than 10% of participants reported facing discrimination indicating general improvements in societal attitudes towards HIV and the stigmas which were associated with it in the past. There was also no clear association of MV rate with the type of caregiver at baseline or socio-economic status. Lack of association with the type of caregiver could simply be because 97% of the participants were infant mothers.

## Limitations

We measured non-attendance at scheduled study visits, with a particular focus on the 18-month time point, and used these study visits as a proxy for attendance at routine postnatal visits. However, we do not have data to confirm that scheduled study visits coincided with routine postnatal visits. Given that we offered infant HIV testing at each time point, and thus earlier referral into care, we believe that this limitation was diluted by the offer of earlier infant HIV diagnoses, as each visit could be a proxy for attendance or uptake of earlier infant HIV testing, a critical component of preventative child health programmes in high HIV prevalence settings. The lack of routine tracking systems did not allow us to establish whether a child who missed their study visit attended a different health facility for their routine child health visit. Our findings raise the importance of patient tracking systems, across rural and urban settings to ensure that no mother-baby pair is lost from care.

The PMTCT regimen changed from Option A to Option B guidelines mid-way of the cohort study, in April 2013, which was the last month of baseline (6-weeks post-partum) enrolment of participants. Although we investigate association of MV with only baseline (including antenatal experiences) characteristics, which were all under the same regimen (Option A), follow-up to study end-points over-lapped with Option B guidelines. The data collection process did not include individual-level information on the specific time that they were moved from Option A to Option B. For this reason, it was not possible to accurately disentangle, nor adjust in the regression models, the effects of treatment policy change on MV, if any.

Recent South African studies, following the introduction (in 2015) of birth PCR HIV testing in infants, have demonstrated poorer infant follow-up postnatally among infants tested at birth [29, 30]. Therefore the retention seen in this study may be an over-estimate of true current retention.

## Conclusions

In this nationally-representative sample, we studied non-attendance at scheduled study visits where we offered additional HIV testing (at 3, 6, 9, 12 and 15 months postpartum) and routine HIV testing at the final PMTCT endpoint of 18-months. Despite us offering these services, which facilitate earlier access to care, we demonstrate that > 50% HEU did not attend one or more scheduled study visits. Poor accessibility of clinics, young age, poor maternal uptake of ART at 6 weeks postpartum and child healthcare unawareness were risk factors for non-attendance at the scheduled study visits during the era of PMTCT Option A and B PMTCT regimens. On-going exploration is needed to understand and remove the barriers for postnatal care uptake in the current era of lifelong ART. Strengthening community-based health care linkages with the facilities could be considered to facilitate tracing and ease of access if similar challenges persist.

## Additional files

Additional file 1: Baseline characteristics at 6 weeks postpartum presented by patterns of MV per time-point. MV- non-attendance at scheduled postnatal care visits. Proportions of participants who missed each follow-up visit point when they were eligible to attend it. The proportions are presented per sub-group of each baseline characteristic measured at 6 weeks postpatrum, i.e., row percentages are given. Significant chi-squared ps for differences in proportions of MV between sub-groups of each baseline characteristic, per visit point, are presented in bold face. All proportions were adjusted for survey sampling weights (W %). South African provinces- WC-Western Cape, NW-North West, NC-Northen Cape, MP-Mpumalanga, LP-Limpopo, KZN-KwaZulu Natal, GP-Gauteng, FS-Free State, EC-Eastern Cape. (XLSX 17 kb)

Additional file 2: Actual number of missed visits (MV-frequency) presented according to the number of scheduled (exposed) visits. The number and percentage of participants who missed a specific number of visits out of the total number of scheduled visits they were expected to attend (exposed visits). The description of ‘exposed visits’ is given in Methods under ‘*Describing patterns of ‘missed visits”* sub-heading*.* For example, a total of 23 participants were expected to have attended only 3 visits (3 was their total number of exposed visits), 7 of these attended all the three visits (MV-frequency = 0) and 16 missed 1 visit. (PDF 53 kb)

## Data Availability

The data are bound by ethical and legal restrictions. To access the data of the South Africa’s Prevention of Mother to Child Transmission Effective study, investigators who are not part of the study team should submit a concept proposal to AG (South Africa Medical Research Council (MRC), principal investigator), Debra Jackson (University of the Western Cape/UNICEF, principal investigator) and Thu-Ha Dinh, MD, MS (US Centers for Disease Control and Prevention (CDC), principal investigator) for approval. Investigators with an approved concept proposal must apply for guest researcher status to obtain access to a workstation and the data. Additionally, they will need to complete data security and confidentiality training, and to sign data use and non-disclosure agreements. The data are not yet available in a stable public repository. Researchers who meet criteria to access the data should contact the author AG at Ameena.Goga@ mrc.ac.za.

## Abbreviations

AIDS: Acquired immunodeficiency syndrome
aIRR: Adjusted incidence rate ratio
aOR: Adjusted odds ratio
ART: Antiretroviral therapy
CI: 95% Confidence intervals
EIA: Enzyme-linked immunosorbent assay
GEE: Generalised estimation equations
HEU: HIV-exposed uninfected
HIV: Human immunodeficiency virus
IMCI: Integrated Management of Childhood Illness
MTCT: Mother-to-child transmission of HIV
PCR: Polymerase chain reaction
PMTCT: Prevention of mother-to-child transmission of HIV

## References

[1] Koller M, Patel K, Chi BH, Wools-Kaloustian K, Dicko F, Chokephaibulkit K, Chimbetete C, Avila D, Hazra R, Ayaya S (2015). Immunodeficiency in children starting antiretroviral therapy in low-, middle-, and high-income countries. J Acquir Immune Defic Syndr.

[2] Sibanda EL, Weller IV, Hakim JG, Cowan FM (2013). The magnitude of loss to follow-up of HIV-exposed infants along the prevention of mother-to-child HIV transmission continuum of care: a systematic review and meta-analysis. AIDS.

[3] Kalembo FW, Zgambo M (2012). Loss to Followup: a major challenge to successful implementation of prevention of mother-to-child transmission of HIV-1 programs in sub-Saharan Africa. ISRN AIDS.

[4] Woldesenbet SA, Jackson D, Goga AE, Crowley S, Doherty T, Mogashoa MM, Dinh TH, Sherman GG (2015). Missed opportunities for early infant HIV diagnosis: results of a national study in South Africa. J Acquir Immune Defic Syndr.

[5] Goga AE, Dinh TH, Jackson DJ, Lombard C, Delaney KP, Puren A, Sherman G, Woldesenbet S, Ramokolo V, Crowley S (2015). First population-level effectiveness evaluation of a national programme to prevent HIV transmission from mother to child, South Africa. J Epidemiol Community Health.

[6] Dramowski A, Coovadia A, Meyers T, Goga A (2011). Identifying Missed Opportunities For Early Intervention Among Hivinfected Paediatric Admissions At Chris Hani Baragwanath Hospital, Soweto, South Africa. South Afr J HIV Med.

[7] World Health Organization (2012). A short guide on methods: measuring the impact of national pmtct programmes: towards the elimination of new HIV infections among children by 2015 and keeping their mothers alive.

[8] Goga AE, Dinh TH, Jackson DJ, Lombard CJ, Puren A, Sherman G, Ramokolo V, Woldesenbet S, Doherty T, Noveve N (2016). Population-level effectiveness of PMTCT option a on early mother-to-child (MTCT) transmission of HIV in South Africa: implications for eliminating MTCT. J Glob Health.

[9] World Health Organization. Antiretroviral drugs for treating pregnant women and preventing HIV infection in infants: recommendations for a public health approach - 2010 version: World Health Organization; 2010. https://www.who.int/hiv/pub/mtct/antiretroviral2010/en/. 26180894

[10] Smits J, Steendijk R (2015). The international wealth index (IWI). Soc Indic Res.

[11] Hilbe JM (2011). Negative Binomial Regression.

[12] Deb P, Trivedi PK (2006). Maximum simulated likelihood estimation of a negative binomial regression model with multinomial endogenous treatment. Stata J.

[13] Department of Health South Africa; South African National AIDS Council: Clinical Guidelines: PMTCT (Prevention of Mother-to-Child Transmission). 2010. https://sahivsoc.org/Files/NDOH_PMTCT%20Apr%202008.pdf.

[14] Godfrey W, Berhan AY, Kudiabor K, Mukaminega M, On’gech J, Nyirabahizi E, Chouraya C, Kimosop D, Ndatimana D, Phiri M (2015). A secondary analysis of retention across the PMTCT cascade in selected countries: Rwanda, Malawi, Kenya, and Swaziland, HIVCore Report.

[15] Merdekios B, Adedimeji AA (2011). Effectiveness of interventions to prevent mother-to-child transmission of HIV in southern Ethiopia. Int J Women's Health.

[16] Mitiku I, Arefayne M, Mesfin Y, Gizaw M (2016). Factors associated with loss to follow-up among women in option B+ PMTCT programme in Northeast Ethiopia: a retrospective cohort study. J Int AIDS Soc.

[17] Tweya H, Gugsa S, Hosseinipour M, Speight C, Ng'ambi W, Bokosi M, Chikonda J, Chauma A, Khomani P, Phoso M (2014). Understanding factors, outcomes and reasons for loss to follow-up among women in option B+ PMTCT programme in Lilongwe, Malawi. Trop Med Int Health.

[18] Kaplan R, Orrell C, Zwane E, Bekker LG, Wood R (2008). Loss to follow-up and mortality among pregnant women referred to a community clinic for antiretroviral treatment. AIDS.

[19] Horwood C, Butler LM, Haskins L, Phakathi S, Rollins N (2013). HIV-infected adolescent mothers and their infants: low coverage of HIV services and high risk of HIV transmission in KwaZulu-Natal, South Africa. PLoS One.

[20] Sam-Agudu NA, Folayan MO, Ezeanolue EE (2016). Seeking wider access to HIV testing for adolescents in sub-Saharan Africa. Pediatr Res.

[21] Fatti G, Shaikh N, Eley B, Jackson D, Grimwood A (2014). Adolescent and young pregnant women at increased risk of mother-to-child transmission of HIV and poorer maternal and infant health outcomes: a cohort study at public facilities in the Nelson Mandela Bay metropolitan district, eastern cape, South Africa. S Afr Med J.

[22] UNAIDS, UNICEF, World Health Organization: HIV prevention among adolescent girls and young women: putting HIV prevention among adolescent girls and young women on the fast-track and engaging men and boys. 2016. https://www.unaids.org/sites/default/files/media_asset/UNAIDS_HIV_prevention_among_adolescent_girls_and_young_women.pdf.

[23] Sidze LK, Faye A, Tetang SN, Penda I, Guemkam G, Ateba FN, Ndongo JA, Nguefack F, Texier G, Tchendjou P (2015). Different factors associated with loss to follow-up of infants born to HIV-infected or uninfected mothers: observations from the ANRS 12140-PEDIACAM study in Cameroon. BMC Public Health.

[24] Technau KG, Kalk E, Coovadia A, Black V, Pickerill S, Mellins CA, Abrams EJ, Strehlau R, Kuhn L (2014). Timing of maternal HIV testing and uptake of prevention of mother-to-child transmission interventions among women and their infected infants in Johannesburg, South Africa. J Acquir Immune Defic Syndr.

[25] Amoran OE, Oluwole FA, Salami OF (2012). A comparative analysis of teenagers and older pregnant women in the utilization of prevention of mother to child transmission [PMTCT] services in, Western Nigeria. BMC Int Health Hum Rights.

[26] Geng EH, Nash D, Kambugu A, Zhang Y, Braitstein P, Christopoulos KA, Muyindike W, Bwana MB, Yiannoutsos CT, Petersen ML (2010). Retention in care among HIV-infected patients in resource-limited settings: emerging insights and new directions. Curr HIV/AIDS Rep.

[27] Wachira J, Naanyu V, Genberg B, Koech B, Akinyi J, Kamene R, Ndege S, Siika AM, Kimayo S, Braitstein P (2014). Health facility barriers to HIV linkage and retention in Western Kenya. BMC Health Serv Res.

[28] Gourlay A, Birdthistle I, Mburu G, Iorpenda K, Wringe A (2013). Barriers and facilitating factors to the uptake of antiretroviral drugs for prevention of mother-to-child transmission of HIV in sub-Saharan Africa: a systematic review. J Int AIDS Soc.

[29] Dunning L, Kroon M, Fourie L, Myer L (2016). Impact of birth HIV PCR testing on routine 6-week EID testing in Cape Town, South Africa. 8th International Workshop on HIV Pediatrics.

[30] Kalk E, Kroon M, Boulle A, Osler M, Euvrard J, Stinson K, Timmerman V, Davies M (2016). Does HIV DNA-PCR testing of HIV-exposed infants at birth reduce follow-up for routine testing at 4-14 weeks of age?. 8th International Workshop on HIV Pediatricsl.

