# Supplementary figures and images for: Factors associated with non-attendance at scheduled infant follow-up visits in an observational cohort of HIV-exposed infants in South Africa, 2012–2014
Source: BMC Infect Dis. 2019 Sep 16;19(Suppl 1):788. doi: 10.1186/s12879-019-4340-5 (PMC6745773; doi:10.1186/s12879-019-4340-5)

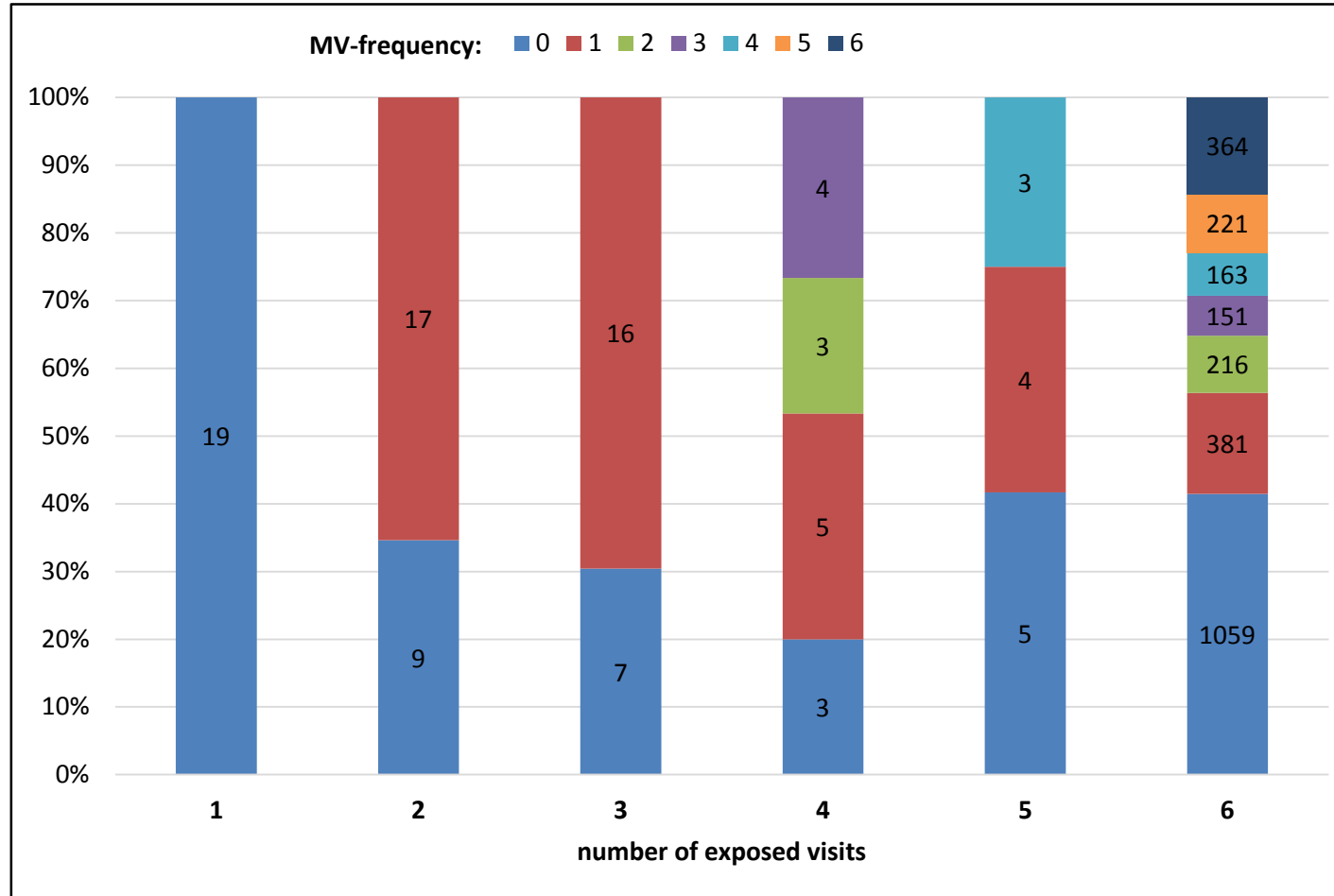

Supplement: Supplementary file 2 — Actual number of missed visits (MV-frequency) presented according to the number of scheduled (exposed) visits. The number and percentage of participants who missed a specific number of visits out of the total number of scheduled visits they were expected to attend (exposed visits). The description of ‘exposed visits’ is given in Methods under ‘Describing patterns of ‘missed visits” sub-heading. For example, a total of 23 participants were expected to have attended only 3 visits (3 was their total number of exposed visits), 7 of these attended all the three visits (MV-frequency = 0) and 16 missed 1 visit. (PDF 53 kb) [file 12879_2019_4340_MOESM2_ESM.pdf]
